# Occurrence of eating difficulties in children self-reported by parents

**DOI:** 10.1590/2317-1782/e20240118en

**Published:** 2025-10-20

**Authors:** Vanessa Souza Gigoski de Miranda, Juliana dos Santos Luiz, Lisiane De Rosa Barbosa, Gilberto Bueno Fischer

**Affiliations:** 1 Programa de Pós-graduação de Pediatria, Universidade Federal de Ciências da Saúde de Porto Alegre – UFCSPA - Porto Alegre (RS), Brasil.; 2 Departamento de Fonoaudiologia, Universidade Federal de Ciências da Saúde de Porto Alegre – UFCSPA - Porto Alegre (RS), Brasil.

**Keywords:** Eating Behavior, Child, Occurrence, Eating Disorders in Childhood, Brazil

## Abstract

**Purpose:**

To estimate the occurrence and characteristics of eating difficulties in Brazilian children.

**Methods:**

Cross-sectional study, carried out from January to June 2022 through the completion of online forms and scales by parents and/or guardians of Brazilian children aged 6 months to 6 years and 11 months old.

**Results:**

The sample included 596 participants, the majority (54.5%) of whom were male, with a gestational age at birth of an average of 38 weeks (SD = 3.07). Eating difficulties were present in 26% of the sample, being more prevalent in males (59.36%). There were positive associations between family education and the presence and severity of eating difficulties. The presence of at least one disease or comorbidity was reported in 59.1% (n = 352) of the sample, with severe eating difficulties being associated with the presence of at least one of the mentioned comorbidities. Children diagnosed with autism spectrum disorder were significantly associated with severe eating difficulties (p = <0.001). The presence of some syndrome or genetic disease was associated (p = 0.021) with moderate eating difficulties. There was a significant association (p = 0.026) between the presence of mild and moderate eating difficulties and the presence of more than one hospitalization.

**Conclusion:**

The occurrence of eating difficulties in Brazilian children is 26%, the majority of which are severe and associated with pre-existing comorbidities.

## INTRODUCTION

Food empowers the human body for growth and development and becomes the channel through which rules and bonds can be transmitted^(1)^. This action is a process of interaction and pleasure, connecting it to emotional, social, cognitive, affective experiences, and learning. It is also directly linked to physical and mental health, requiring the integrity of the sensory systems and other functions involved in the digestive system. Thus, alterations in any of these areas predispose to the development of eating disorders^(2)^.

Culturally, it is believed that eating is a reflexive act of the human being. However, it is a habit learned from birth and is based on social, sensory, and physical experiences^(3)^. Feeding difficulties (FDs) can be defined as impaired oral intake that is age not compromised for and is associated with medical, nutritional, feeding, and/or psychosocial dysfunction^(4)^, which can affect family relationships^(5)^. Furthermore, they can have serious consequences for children, such as failure to thrive, developmental delay, and risk of death^(6)^. Studies indicate that FD affects approximately 8% to 50% of children, regardless of age, gender, ethnicity, and economic status. The prevalence varies between 25% and 35% among children with typical development, and can reach up to 80% in children with developmental disabilities^(7,8)^. For families, FDs represent one of the most important concerns faced in childhood, being a frequent cause of conflict in relationships between parents and children^(9)^.

According to Junqueira^(10)^, risk factors for the development of FDs include acute and/or chronic health conditions, gastrointestinal pathologies, food allergies, cardiac system dysfunctions and diseases, oral motor system disability or dysfunction, changes in sensory system integration, and conflicting emotions. Despite this, studies identify that 30% of children without any of these risk factors will experience some challenge during the feeding process^(11)^. While feeding difficulties are common in pediatric practice and are a common complaint from parents and guardians in pediatric speech-language pathology clinics, studies and research in this area become essential given the lack of studies estimating the occurrence of HIs in children in the Brazilian population. Therefore, it is important to verify the occurrence of FDs in the Brazilian population to subsequently discuss treatments for these difficulties, enabling accurate and effective referrals. The objective of this study is to estimate the occurrence of HIs in Brazilian children, as self-reported by parents, and to characterize this population.

## METHOD

This is a cross-sectional study, approved by the Research Ethics Committee of the Santo Antônio Children's Hospital of the Irmandade Santa Casa de Misericórdia Hospital Complex in Porto Alegre, under opinion No. 4,552,335/2021, and conducted from January to June 2021. The survey was publicized through social media posts with a QR code that directed interested parties to the questionnaire for completion.

The baseline sample consisted of parents and/or guardians of children who met the inclusion criteria: Brazilian infants and children residing in the country, aged between 6 months and 6 years and 11 months, considering the minimum corrected age of 6 months for the initiation of solid food introduction, and who had already had at least one oral feeding experience. Participants who did not complete the questionnaire completely were excluded. Parents or guardians who felt comfortable answering questions about their children were required to sign an Informed Consent Form (ICF) to continue and complete the questionnaire. This questionnaire consisted of four items developed by the researchers: child characteristics and health (such as sex, race, age, type of delivery, hospital discharge, presence of comorbidities, history of hospitalizations, admissions, and intubations, breastfeeding, use of bottles and nipples, initiation of solid foods, and feeding route and consistency at the time of collection), characteristics of the guardian (such as education and occupation), socioeconomic information (such as family income), and the Brazilian Infant Feeding Scale (BIFS)^(12)^.

The BIFS is a self-report instrument, completed by parents and/or caregivers, which contains 14 questions regarding the child's feeding times and how caregivers feel about these identified behaviors. Finally, the results can be converted into scores, resulting in an outcome—the presence or absence of feeding difficulties—and, if present, a severity level (mild, moderate, or severe) can be determined. The questionnaire responses were analyzed by a team of two speech-language pathologists, and the results were standardized to standardize the researchers' use and analysis of the BIFS screening instrument.

The database was created using Google Sheets. Descriptive and qualitative analyses were performed, presented as absolute and relative frequencies. The results of quantitative variables were presented as symmetric variables using mean and standard deviation, and asymmetric variables were presented as median and interquartile range (IQR). Normality was verified using the K-S test. To assess associations with BIFS, the chi-square test was applied, using adjusted standardized residuals, ANOVA, and Kruskal-Wallis, with Bonferroni correction for multiple comparisons. The significance level adopted was 0.05 and the analyses were performed using the SPSS statistical software (IBM SPSS Statistics for Windows, Version 25.0. Armonk, NY: IBM Corp.).

## RESULTS

Table 1 presents the characteristics of the population that responded to the questionnaire about their children's nutrition and development. The sample included 596 parents and guardians. Regarding their educational level, the majority (74%) had higher education, and only 1.3% (n = 8) reported having incomplete elementary education. Regarding the caregivers' occupations, the majority (60.2%) reported being salaried. Regarding the reported family income, the majority (69.5%) reported earning more than three minimum wages, and only 3.2% (n = 19) earned less than one minimum wage.

**Table 1:** Characterization of parents/caregivers

Characteristics		n (%)
Education	Higher Education	441 (74.0)
	Incomplete Elementary	8 (1.3)
	Complete Elementary	10 (1.7)
	Incomplete high school	9 (1.5)
	Complete high school	128 (21.5)
Profession	Employed Self-	359 (60.2)
	Employed/Informal	187 (31.4)
	Unemployed	50 (8.4)
Family Income	More than 3 minimum wages	414 (69.5)
	Less than 1 minimum wage	19 (3.2)
	1 minimum wage	47 (7.9)
	1-2 minimum wages	114 (19.1)
	NI	2 (0.3)

**Caption:** NI: not informed.

Table 2 presents the characteristics of the children self-reported by the parents or guardians. The majority of the sample was male (54.5%). Regarding the need for hospitalizations, the majority (75.2%) of the sample reported no hospitalizations after hospital discharge, while 24.8% (n = 148) reported needing hospitalization at least once after hospital discharge after birth.

**Table 2:** Characterization of children self-reported by parents and caregivers

Characteristics		n (%)
Sex	Masculine	325 (54.5)
	Feminine	271 (45.5)
Gestational Age (weeks)	Mean ± SD	38 ± 3.07
Current Age (weeks)	Median [IQR]	30 [17;47]
Childbirth	Normal	216 (36.2)
	Cesarean Section	380 (63.8)
Has your child ever been hospitalized?	Yes	148 (24.8)
	No	448 (75.2)
Number of hospitalization	Nothing	448 (75.2)
	One	94 (15.8)
	More one	50 (8.4)
	NR	4 (0.7)
Have you ever needed to be intubated (orotracheal intubation)	Yes	35 (5.9)
	No	561 (94.1)
Have required more than one episode of intubation (n=35)	Yes	16 (45.7)
	No	19 (54.3)

**Caption:** SD: Standard derivation; IR = interquartile range; NR: not reported

**Figure 1:**
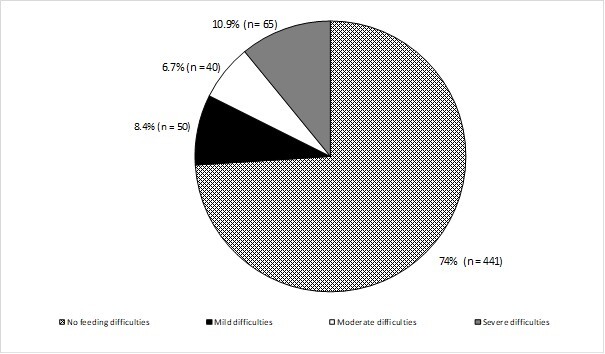
Severity of BIFS Outcome

Figure 1 shows the severity of the BIFS outcome. Some degree of FD was present in 26% (n = 155) of the sample, while the majority (74%) did not present any FD. Of the individuals with FDs identified by the instrument, 65 participants (10.9%) were diagnosed with severe FDs, 40 (6.7%) had moderate difficulty, and 50 (8.4%) had mild difficulty.

Of the 155 individuals who presented with some degree of FDs, the majority (59.36%) were male. Of the children who had FDs, 41 (27.15%) were reported to have been hospitalized at least once. A significant association was found between the presence of mild and moderate FDs and the presence of more than one hospitalization in these children (p = 0.026).

Regarding the associative analyses of the BIFS, based on the analysis using the chi-square test with the aid of adjusted standardized residuals, ANOVA, and Kruskal-Wallis with Bonferroni correction for multiple comparisons, a significant association was found between older children and the presence of mild FD, compared to children without difficulties (p = 0.030).

Regarding the family characteristics of the sample, there was a positive association between family education and the presence and severity of FD. Families with a high school diploma were associated with severe FD (p = <0.001), while families with a college degree were associated with the outcome of no FD. Regarding family income, there was a positive association with FD severity. Children without FD in the BIFS outcome were from families earning more than three minimum wages (p = <0.001), children with mild difficulties were from families earning between one and two minimum wages, and moderate and severe BIFS outcomes were associated with families of children earning up to one minimum wage.

**Figure 2:**
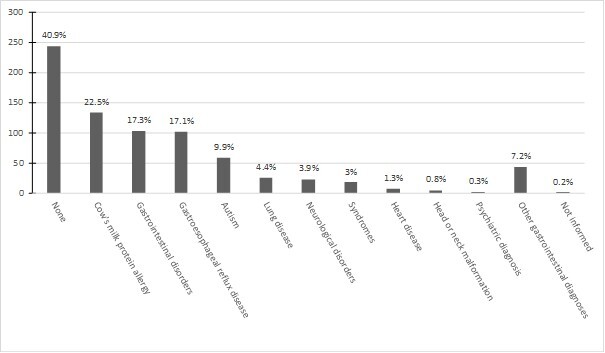
Associated comorbidities in the sample

Regarding the presence of diseases or comorbidities in the sample, these included gastroesophageal reflux disease, cow's milk protein allergy, or other gastrointestinal disorders; head or neck malformation, autism, neurological disorder, heart disease, lung disease, psychiatric diagnosis, or the presence of a syndrome or other genetic disorder, the presence of at least one of these was reported in 59.1% (n = 352) of the sample. The distribution of each comorbidity is shown in Figure 2.

Regarding the presence of these associated comorbidities (such as cow's milk protein allergy, gastrointestinal disorders, gastroesophageal reflux disease, physiological gastroesophageal reflux, autism, lung disease, neurological disorders, syndromes, heart disease, head or neck malformation, psychiatric diagnosis, and others), the outcome of severe FD was associated with their presence, while children without HL showed a negative association with the comorbidities item (p = 0.002). Among the diseases and disorders reported by parents in the instrument, children diagnosed with autism spectrum disorder (n = 29) were significantly associated (p = <0.001) with severe FD. The presence of some syndrome or genetic disease was associated (p = 0.021) with moderate FD in this sample. Children diagnosed with congenital heart disease (n = 5) showed a significant association (p = 0.016) with the outcome of mild FD.

## DISCUSSION

Childhood eating disorders can produce qualitative and/or quantitative manifestations in the feeding process and may originate from multifactorial conditions^(1,7,11,13-17)^. Some degree of eating disorders was present in 26% (n=155) of the sample that responded to the questionnaire. The BIFS^(12)^, the instrument used to estimate this incidence, is a screening tool that can be used by any healthcare professional and serves to track the presence and severity of childhood eating disorders. However, it is not an assessment tool that addresses the cause and treatment direction. A comprehensive, multidisciplinary evaluation should be performed whenever possible. Conducting an adequate assessment of feeding difficulties in infants and children is essential to determine the need for referrals, select appropriate therapeutic approaches, and monitor treatment effectiveness^(18)^. Effective assessment and treatment of Pediatric Feeding Disorders (PFD) require the involvement of multiple professions and areas of expertise.

A similar gender distribution was found in the overall study sample, but a higher incidence of boys with feeding difficulties was observed. This finding is similar to a study of children aged 0 to 4 years^(19)^, which observed a higher incidence of FD in boys, and a 2020 Brazilian study^(19)^, which identified 65.1% of the sample of boys aged 0 to 10 years with FDs.

A significant association between older children and the presence of mild FD, compared to children without difficulties, was found in this study, which may be explained by the environmental influences to which children are exposed throughout their development. In a systematic review^(1)^, it was found that the predominant age group for children with FD is preschoolers. The average age found in a Brazilian study^(20)^ of children diagnosed with food selectivity is higher than that found in the present study, at 52.92 months. However, in that same study, the average diagnosis of "limited appetite" was 31.83, which is close to the median age of children with FDs found in our study. The term refers to one of the classifications of children with eating difficulties, which, according to the reference center that produced the study, children with FDs were divided into: food selectivity, reduced appetite, and food phobia. This finding can be explained by the aforementioned prevalence study^(20)^ being conducted with children across a wide age range (0 to 10 years), while our study included children aged 6 months to 6 years and 11 months. Furthermore, the previous study did not use validated diagnostic instruments, unlike the current study.

Regarding family socioeconomic variables, there was a higher incidence of families with higher education and higher income. This finding may be explained by the fact that this study was developed through an online questionnaire and disseminated through social media, which promotes greater access for families with higher education and higher per capita income^(20)^. In this study, there was an association between severe FD and families with a high school diploma, while families with a college diploma were associated with the outcome without FD. It was found that the higher the parental education, the lower the incidence of FDs. This finding may be explained by greater access to quality information and a qualified multidisciplinary team from birth^(21)^.

Regarding family income, there was a positive association with the severity of FD, with children without FD at the BIFS outcome belonging to families earning more than three minimum wages, and moderate and severe BIFS outcomes were associated with families of children earning up to one minimum wage. In the literature^(22)^, a higher incidence of FDs was identified in families with a monthly income above two minimum wages, which differs from the findings of this study, as well as of a Dutch study^(23)^, which observed an association between food selectivity and children with lower family income. In a cohort that followed 4-year-old children diagnosed with "fussy" and "picky" eating behavior (defined by researchers as an eating behavior profile characterized by high food fussiness, slow eating, and a satiety response, combined with low enjoyment of food and response to food), there was also an association with family income, in which fussy and picky behavior at mealtimes was more frequent in low-income families than in non-fussy ones (42% versus 31.8%, respectively)^(23)^.

Regarding the presence of diseases or comorbidities, they were found in 59.1% (n = 352) of the sample, with gastrointestinal disorders and food allergies being the most frequent. Junqueira^(10)^ states that pathologies of the gastrointestinal system present as organic health conditions that need to be identified and treated due to the repercussions and negative association internalized in the child with the task of eating; and the presence of pain associated with mealtimes. In the sample, the presence of comorbidities was associated with severe FDs, and the absence of comorbidities with the outcome without FDs, corroborating findings in the literature that indicate the presence of comorbidities is associated with a higher occurrence of FDs^(10,11,24)^. Among the associations evidenced in this study, autism spectrum disorder, the presence of a genetic syndrome or disease, and a diagnosis of congenital heart disease were associated with some level of severity of FDs. This may be explained by these children's higher risk of FDs, such as refusal and selectivity of certain foods due to oral-motor dysfunctions, behavioral problems, and sensory processing disorders^(25,26)^.

Despite this association with comorbidities present in the sample, only 27.15% of the sample of children with FDs had been hospitalized after discharge from the hospital, and these were significantly associated with mild and moderate FD outcomes. The relationship between hospitalizations and FDs can be explained by the aversive experiences children experience in hospital settings, such as intensive care unit stays, use of alternative feeding routes and mechanical ventilation, and airway aspirations, among others^(27)^. These factors can contribute to increased oral sensitivity, which results in an aversion to new food textures^(28)^.

The instrument used to identify FDs and their severity, the BIFS^(11)^, uses parents' perceptions of their child's eating problems. Instruments of this type are highly relevant, as parents/caregivers, by observing numerous meals, can gain a broader perspective on their child's typical eating behavior. Therefore, their reports can be an applicable and reliable form of assessment, providing important information about typical mealtime behavior and skills, including mealtime^(12)^.

The present study has limitations due to its observational nature, using a screening instrument rather than comprehensive case assessments. It is worth noting that there is currently no validated assessment instrument for FDs in the Brazilian literature. Another limitation was that it was developed using an online questionnaire, which can generate selection bias and ultimately select individuals with higher income and education levels. However, the findings of this study contribute to a better understanding of the topic of FDs, which are rarely found in the literature, especially in Brazil. Furthermore, this is one of the few occurrence studies in Brazilian literature, proving innovative for using a validated instrument to determine the presence or absence of FDs and their severity. Therefore, further studies addressing the occurrence, incidence, diagnosis, and treatment of FDs are needed.

## CONCLUSION

Therefore, it was possible to estimate the incidence of feeding difficulties among Brazilian children in this study, based on the completion of the BIFS instrument by parents or guardians, at 26%. Most children with FDs had severe severity, associated with pre-existing comorbidities. There was a positive association between education level and family income and the presence and severity of FD.
