# Supplementary material for: Occurrence of eating difficulties in children self-reported by parents
Source: Codas. 2025 Oct 20;37(5):e20240118. doi: 10.1590/2317-1782/e20240118en (PMC12617281; doi:10.1590/2317-1782/e20240118en)
Supplement: Translation

# Translation: Ocorrência das dificuldades alimentares em crianças autorreportada pelos pais

**DOI:** 10.1590/2317-1782/e20240118pt

**Authors:** Vanessa Souza Gigoski de Miranda, Juliana dos Santos Luiz, Lisiane De Rosa Barbosa, Gilberto Bueno Fischer

**Affiliations:** 1 Programa de Pós-graduação de Pediatria, Universidade Federal de Ciências da Saúde de Porto Alegre – UFCSPA - Porto Alegre (RS), Brasil.; 2 Departamento de Fonoaudiologia, Universidade Federal de Ciências da Saúde de Porto Alegre – UFCSPA - Porto Alegre (RS), Brasil.

**Keywords:** Comportamento Alimentar, Criança, Ocorrência, Transtornos de Alimentação na Infância, Brasil

## Abstract

**Objetivo:**

Estimar a ocorrência e características das dificuldades alimentares em crianças autorreportada pelos pais.

**Método:**

Estudo transversal, realizado de janeiro a junho de 2022 através do preenchimento de formulários e escalas online por pais e/ou responsáveis por crianças brasileiras de 6 meses a 6 anos e 11 meses de idade.

**Resultados:**

A amostra contou com 596 participantes, sendo a maioria (54,5%) do sexo masculino, com idade gestacional ao nascimento em média de 38 semanas (DP = 3.07). Dificuldades alimentares se mostraram presentes em 26% da amostra, sendo mais prevalentes nos indivíduos do sexo masculino (59,36%). Houve associações positivas entre a escolaridade familiar e a presença e gravidade das dificuldades alimentares. A presença de pelo menos uma doença ou comorbidade foi referida em 59,1% (n = 352) da amostra, sendo as dificuldades alimentares severas associadas a presença de pelo menos uma das comorbidades referidas. Crianças com diagnóstico de transtorno do espectro autista foram associadas significativamente com dificuldades alimentares severas (p = <0.001). A presença de alguma síndrome ou doença genética foi associada (p = 0,021) a dificuldade alimentar moderada. Verificou-se associação significativa (p = 0,026) entre a presença de dificuldades alimentares leves e moderadas com a presença de mais de uma hospitalização.

**Conclusão:**

A ocorrência de dificuldades alimentares nas crianças brasileiras desse estudo é de 26%, sendo a maioria com gravidade severa, estando associada a comorbidades pré-existentes.

## INTRODUÇÃO

A alimentação potencializa o corpo humano ao crescimento e desenvolvimento, e se torna a via pela qual regras e vínculos podem ser transmitidos^(1)^. Tal ação é um processo de interação e prazer, estando conectada a experiências emocionais, sociais, cognitivas, afetivas e ao aprendizado. Ela também está diretamente ligada à saúde física e mental, demandando a integridade dos sistemas sensoriais e outras funções envolvidas no sistema digestório. Desta forma, alterações em uma dessas áreas predispõe ao desenvolvimento de alterações alimentares^(2)^.

Culturalmente acredita-se que comer é um ato reflexo do ser humano. No entanto, trata-se de um hábito aprendido a partir do nascimento e baseia-se em experiências sociais, sensoriais e físicas^(3)^. As dificuldades alimentares (DAs) podem ser definidas como uma ingestão oral prejudicada que não é apropriada para a idade e está associada a disfunção médica, nutricional, de alimentação e/ou psicossocial^(4)^, podendo afetar as relações familiares^(5)^. Além disso, podem apresentar consequências graves para as crianças, como: falha no crescimento, atraso no desenvolvimento e risco de óbito^(6)^. Estudos apresentaram dados de que a DA acomete cerca de 8% a 50% das crianças independentemente de idade, sexo, etnia e condição econômica, sendo a incidência variável entre 25% e 35% entre crianças que apresentam um desenvolvimento típico, podendo chegar ematé 80% em crianças com alteração de desenvolvimento^(7,8)^. Para a família, as DAs representam uma das preocupações mais importantes enfrentadas na infância, sendo causa frequente de conflitos nas relações entre pai, mãe e filhos^(9)^.

Segundo Junqueira^(10)^, dentre os fatores de risco para o desenvolvimento de DAs estão: as condições de saúde agudas e/ou crônicas, patologias do sistema gastrintestinal, alergias alimentares, disfunções dos sistemas cardíaco e respiratório, inabilidade ou disfunções do sistema motor oral, alterações de integração dos sistemas sensoriais e emoções conflituosas. Apesar disso, estudos identificam que 30% das crianças sem nenhum desses fatores de risco terão algum desafio frente ao processo de alimentação^(11)^. Ao passo que dificuldades na área de alimentação são frequentes na prática pediátrica e concretizam-se como uma queixa dos pais e responsáveis na clínica fonoaudiológica infantil, estudos e pesquisas nesta área tornam-se imprescindíveis quando também observa-se a falta de estudos que estimem a ocorrência das DAs nas crianças da população brasileira. Dessa forma, torna-se importante verificar a ocorrência das DAs na população brasileira para, posteriormente, se discutir sobre tratamentos dessas dificuldades, possibilitando encaminhamentos precisos e eficazes. O objetivo desse estudo é de estimar a ocorrência de DAs em crianças brasileiras autorreportada pelos pais e caracterizar essa população.

## MÉTODO

Trata-se de um estudo transversal, aprovado pelo Comitê de Ética em Pesquisa do Hospital da Criança Santo Antônio do Complexo Hospitalar da Irmandade Santa Casa de Misericórdia de Porto Alegre, sob o parecer nº4.552.335/2021 e desenvolvido no período de janeiro a junho de 2021. A pesquisa foi divulgada através de publicações em redes sociais com *QR Code* que direcionava o interessado ao questionário para preenchimento.

A amostra de base foi composta por pais e/ou responsáveis de crianças que atendessem aos critérios de inclusão: bebês e crianças de nacionalidade brasileira e residentes no país, com idade entre 06 meses a 06 anos e 11 meses, considerando a idade corrigida mínima de 6 meses para início da introdução alimentar, e que já tivessem tido ao menos uma experiência de alimentação por via oral. Foram excluídos participantes que não preencheram de forma completa o questionário. Os pais ou responsáveis que se sentissem à vontade para responder as perguntas sobre as crianças precisavam concordar com o Termo de Consentimento Livre e Esclarecido (TCLE) para prosseguir e responder o questionário. Esse questionário foi composto por 4 itens elaborados pelas pesquisadoras, sendo: caracterização da criança e sua saúde (como sexo, raça, idade, tipo de parto, alta hospitalar, presença de comorbidades, histórico de hospitalizações, internações e intubações, aleitamento materno, uso de mamadeiras e bicos, início da introdução alimentar e via de alimentação e consistência na época da coleta) caracterização do responsável (como escolaridade e profissão), informações socioeconômicas (como a renda familiar), e mais a Escala Brasileira de Alimentação Infantil (EBAI)^(12)^.

A EBAI é um instrumento de autopreenchimento, respondido pelos pais e/ou cuidadores, que apresenta 14 questões referentes ao momento de alimentação da criança e de como os cuidadores se sentem frente a esses comportamentos identificados. Ao fim, é possível realizar uma conversão em escores que resultará em um desfecho - presença ou não de dificuldade alimentar - e, se presente, graduar o nível de severidade (leve, moderado ou severo). A análise das respostas do questionário foi conduzida por uma equipe de duas fonoaudiólogas e houve uma equalização para a padronização das pesquisadoras em relação ao uso e análise do instrumento de triagem EBAI.

O banco de dados foi criado utilizando o *Google Sheets*. Realizou-se uma análise descritiva e qualitativas apresentados através de frequências absoluta e relativa. Já os resultados das variáveis quantitativas, foram apresentadas em variáveis simétricas, através de média e desvio padrão, e as variáveis assimétricas foram apresentadas através de mediana e intervalo interquartil (IQR). A normalidade foi verificada pelo teste K-S. Para verificar as associações com a EBAI, foram aplicados os testes Qui-Quadrado, com auxílio dos resíduos padronizados ajustados, ANOVA e Kruskal-Wallis, com correção de Bonferroni para as comparações múltiplas. O nível de significância adotado foi de 0,05 e as análises foram realizadas no software estatístico SPSS (IBM SPSS Statistics for Windows, Version 25.0. Armonk, NY: IBM Corp.).

## RESULTADOS

A Tabela 1 apresenta as características da população que respondeu ao questionário acerca da alimentação e desenvolvimento de seus filhos. A amostra contou com 596 pais e responsáveis. Quanto à escolaridade deles, a maioria (74%) possuía ensino superior e apenas 1,3% (n = 8) declarou possuir fundamental incompleto. No que tange a profissão dos cuidadores, a maioria (60,2%) declarou-se assalariado. Já em relação à renda familiar referida por estes, a maioria (69,5%) declarou receber mais de 3 salários-mínimos e apenas 3,2% (n = 19) recebiam menos de 1 salário mínimo.

**Tabela 1:** Caracterização dos pais/cuidadores

Características		n (%)
Escolaridade	Superior	441 (74,0)
	Fundamental incompleto	8 (1,3)
	Fundamental completo	10 (1,7)
	Médio incompleto	9 (1,5)
	Médio completo	128 (21,5)
Profissão	Assalariado	359 (60,2)
	Autônomo/Informal	187 (31,4)
	Desempregado	50 (8,4)
Renda familiar	Mais de 3 salários-mínimos	414 (69,5)
	Menos de 1 salário-mínimo	19 (3,2)
	1 salário-mínimo	47 (7,9)
	1-2 salários-mínimos	114 (19,1)
	NI	2 (0,3)

**Legenda:** NI: não informado

Na Tabela 2 são apresentadas as características das crianças autorreportada pelos pais ou responsáveis. A maioria da amostra era do sexo masculino (54,5%). No que diz respeito à necessidade de hospitalizações, a maioria (75,2%) da amostra não reportou nenhuma hospitalização após a alta hospitalar, enquanto 24,8% (n = 148) relatou a necessidade de hospitalização pelo menos uma vez, após a alta hospitalar após o nascimento.

**Tabela 2:** Caracterização das crianças autorreportadas pelos pais e cuidadores

Características		n (%)
Sexo	Masculino	325 (54,5)
	Feminino	271 (45,5)
Idade gestacional (semanas)	Média ± DP	38 ± 3.07
Idade atual (meses)	Mediana [IQR]	30 [17;47]
Tipo de parto	Normal	216 (36,2)
	Cesária	380 (63,8)
Seu filho já teve alguma hospitalização?	Sim	148 (24,8)
	Não	448 (75,2)
Quantidade de hospitalizações	Nenhuma	448 (75,2)
	Uma	94 (15,8)
	Mais de uma	50 (8,4)
	NI	4 (0,7)
Já necessitou ser intubado (intubação orotraqueal)	Sim	35 (5,9)
	Não	561 (94,1)
Já necessitou de mais de um episódio de intubação (n=35)	Sim	16 (45,7)
	Não	19 (54,3)

**Legenda:** DP: desvio padrão; IQR = intervalo interquartil; NI: não informado

A Figura 1 apresenta a gravidade do desfecho da EBAI. Houve presença de DA em algum nível em 26% (n = 155) da amostra, enquanto a maioria (74%) não apresentou nenhuma DA. Dos indivíduos com DAs apontadas pelo instrumento, 65 participantes (10,9%) apresentaram diagnóstico de DA severa, 40 (6,7%) apresentou dificuldade moderada e 50 (8,4%) apresentou dificuldade leve.

**Figura 1:** Gravidade do desfecho da EBAI

Da amostra de 155 indivíduos que apresentaram DAs em algum nível, a maioria (59,36%) eram do sexo masculino. Das crianças que possuíam DAs, 41 (27,15%) foram informadas que haviam sido hospitalizadas pelo menos uma vez. Verificou-se associação significativa entre a presença de DAs leves e moderadas com a presença de mais de uma hospitalização nessas crianças (p = 0,026).

Ainda quanto às análises associativas da EBAI, a partir da análise através da aplicação dos testes Qui-Quadrado com auxílio dos resíduos padronizados ajustados, ANOVA e Kruskal-Wallis com correção de Bonferroni para as comparações múltiplas, encontrou-se associação significativa entre crianças mais velhas e a presença da DA leve, do que as crianças sem dificuldades (p = 0,030).

Quanto às características familiares da amostra, houve associação positiva entre a escolaridade familiar e a presença e gravidade da DA. Famílias com ensino médio completo apresentaram associação com a DA severa (p = <0.001), enquanto as famílias com ensino superior completo, foram associadas ao desfecho de sem DA. Quanto à renda familiar houve associação positiva com a gravidade da DA. Crianças sem DA no desfecho da EBAI pertenciam a famílias que ganhavam acima de 3 salários mínimos (p = <0.001), crianças com dificuldades leves em famílias que ganhavam entre 1 e 2 salários mínimos e, os desfechos moderada e severa da EBAI, foram associadas a famílias de crianças que recebiam até 1 salário mínimo.

Em relação a presença de doenças ou comorbidades na amostra, sendo elas: doença do refluxo gastroesofágico, alergia à proteína do leite de vaca ou outras alterações gastrointestinais; má-formação em cabeça ou pescoço, autismo, alteração neurológica, cardiopatia, doença pulmonar, diagnóstico psiquiátrico, presença de síndrome ou outra alteração genética – a presença de pelo menos uma dessas foi referida em 59,1% (n = 352) da amostra. A distribuição da presença de cada comorbidade está apresentada na Figura 2.

**Figura 2:** Comorbidades associadas na amostra

Quanto à presença dessas comorbidades associadas (como alergia à proteína do leite de vaca, alteração gastrointestinal, doença do refluxo gastroesofágico, refluxo gastroesofágico fisiológico, autismo, doença pulmonar, alteração neurológica, síndromes, cardiopatia, má-formação em cabeça ou pescoço, diagnóstico psiquiátrico e outros), o desfecho de DA severa foi associado a sua presença, enquanto as crianças sem DAs apresentaram associação negativa com o item comorbidades (p = 0,002). Dentre as doenças e transtornos apontadas pelos pais no instrumento, as crianças com diagnóstico de transtorno do espectro autista (n = 29) foram associadas significativamente (p = <0.001) com a DA severa. A presença de alguma síndrome ou doença genética foi associada (p = 0,021) a DA moderada nessa amostra. Crianças com diagnóstico de cardiopatia congênita (n= 5) apresentaram associação significativa (p = 0,016) com desfecho de DA leve.

## DISCUSSÃO

As DAs na infância podem gerar manifestações qualitativas e/ou quantitativas no processo de alimentação podendo ser originárias de condições multifatoriais^(1,7,11,13-17)^. Houve a presença de DAs em algum nível em 26% (n=155) da amostra que respondeu ao questionário. A EBAI^(12)^, instrumento utilizado para alcançar essa estimativa de ocorrência, é um *screening* que pode ser utilizado por qualquer profissional da saúde e tem um papel de rastrear a presença e severidade das DAs na infância, porém, não é um instrumento de avaliação que abordaria causa e direcionamento de tratamento, e uma avaliação completa e multidisciplinar deve ser realizada quando possível. Realizar uma avaliação adequada das dificuldades alimentares de bebês e crianças é imprescindível para determinar a necessidade ou não de encaminhamentos, selecionar as abordagens terapêuticas apropriadas e monitorar a eficácia do tratamento^(18)^. Avaliação e tratamentos efetivos do Distúrbio Alimentar Pediátrico (DAP) requerem o envolvimento de múltiplas profissões e áreas do conhecimento^(18)^.

Verificou-se uma distribuição semelhante de sexo na amostra geral do estudo, porém, uma ocorrência maior de meninos com desfecho com dificuldades alimentares. Tal achado é semelhante a um estudo realizado com crianças de 0 a 4 anos incompletos^(19)^, que observou uma ocorrência de DA superior no sexo masculino, e a um estudo brasileiro de 2020^(19)^ que identificou 65,1% da amostra de meninos de 0 a 10 anos de idade com DAs.

Uma associação significativa entre crianças mais velhas e a presença da DA leve, do que as crianças sem dificuldades, foi encontrada neste estudo, o que pode ser justificado pelas influências ambientais em que estão expostas as crianças com o decorrer do seu desenvolvimento. Em uma revisão sistemática^(1)^, verificou-se que a faixa etária predominante em crianças com DA é a de pré-escolares. A média de idade encontrada em um estudo brasileiro^(20)^, das crianças com diagnóstico de seletividade alimentar, é superior à encontrada no presente estudo, sendo de 52,92 meses. No entanto, nesse mesmo estudo, a média do diagnóstico de “apetite limitado” foi de 31,83, que se aproxima da mediana de idade das crianças com DAs encontradas em nosso estudo. O termo refere-se a uma das classificações das crianças com dificuldades alimentares, que, segundo o centro de referência que produziu o estudo, as crianças com DAs eram divididas entre: seletividade alimentar, apetite reduzido e fobia alimentar. Tal achado pode ser explicado pelo estudo de prevalência anteriormente citado^(20)^ ser realizado com crianças em uma grande faixa etária (0 a 10 anos), enquanto nosso estudo apresenta crianças de 6 meses a 6 anos e 11 meses de idade, bem como, do estudo anterior não utilizar instrumentos de diagnóstico validados, diferente do estudo atual.

Quanto às variáveis socioeconômicas familiares, houve maior ocorrência de famílias com ensino superior completo e maior renda. Tal achado pode ser justificado pelo presente estudo ter sido desenvolvido através de questionário online e divulgado através de redes sociais, o que promove maior acesso de famílias com maior escolaridade e maior renda per capita^(20)^. Nesse estudo, houve associação entre a DA severa e as famílias com ensino médio completo, enquanto as famílias com ensino superior completo, foram associadas ao desfecho de sem a DA, identificando que quanto maior a escolaridade dos pais, menor a ocorrência de DA. Tal achado pode ser justificado pelo maior acesso a informações de qualidade e à equipe multiprofissional qualificada desde o nascimento dessas crianças^(21)^.

Quanto à renda familiar houve associação positiva com a gravidade da DA, em que crianças sem DA no desfecho da EBAI pertenciam a famílias que ganhavam acima de 3 salários mínimos, e os desfechos moderada e severa da EBAI, foram associadas a famílias de crianças que recebiam até 1 salário mínimo. Na literatura^(22)^, foi identificada maior ocorrência das DAs em famílias com renda mensal superior a dois salários-mínimos, o que diverge do encontrado neste estudo, bem como em um estudo holandês^(23)^, em que foi observada a associação entre seletividade alimentar e crianças de menor renda familiar. Em uma coorte que acompanhou crianças de 4 anos com diagnóstico de comportamento alimentar “agitado” e “exigente” (definido pelos pesquisadores como perfil de comportamento alimentar caracterizado por alta exiguidade alimentar, lentidão na alimentação e resposta à saciedade, em combinação com baixo prazer pela comida e resposta à comida), também houve associação com a renda familiar, em que o comportamento agitado e exigente no momento das refeições era mais frequentemente encontrado nas famílias com baixa renda, do que os não exigentes (42% versus 31,8%, respectivamente)^(23)^.

Em relação a presença de doenças ou comorbidades, foi verificada a presença em 59,1% (n = 352) da amostra, sendo as alterações gastrointestinais e alergias alimentares as mais frequentes. Junqueira^(10)^ refere que as patologias do sistema gastrointestinal se apresentam como condições de saúde orgânicas que precisam ser identificadas e tratadas pela repercussão e associação negativa internalizada na criança com a tarefa de alimentação; presença de dor associada aos momentos de refeições. Na amostra relacionou-se a presença de comorbidades com DAs severa e a ausência de comorbidades com o desfecho sem DAs, corroborando com achados da literatura que trazem a presença de comorbidades associadas com a maior ocorrência das DAs^(10,11,24)^. Dentre as associações evidenciadas neste estudo, encontrou-se o transtorno do espectro do autismo, a presença de alguma síndrome ou doença genética, e o diagnóstico de cardiopatia congênita, associados a presença em algum nível de severidade das DAs, o que pode ser explicado por essas crianças terem maior risco de apresentarem DAs, como recusa e seletividade de determinados alimentos, devido a disfunções motoras-orais, problemas comportamentais, e transtornos do processamento sensorial^(25,26)^.

Apesar dessa associação com comorbidades presentes na amostra, somente 27,15% da amostra das crianças com DAs haviam passado por alguma hospitalização após a alta hospitalar do nascimento e essas, associaram-se significativamente com desfechos de DAs leve e moderada. A relação entre a presença de hospitalizações e a presença das DAs pode ser explicada devido às experiências aversivas vivenciadas pelas crianças em ambientes hospitalares como: período de internação em unidade de terapia intensiva, uso de via alternativa de alimentação e de ventilação mecânica, aspirações de vias aéreas, entre outros^(27)^. Tais fatores podem contribuir para o aumento da sensibilidade oral, o qual resulta na aversão a novas texturas alimentares^(28)^.

O instrumento utilizado para identificação das DAs e sua severidade, a EBAI^(11)^, utiliza-se da percepção dos pais acerca dos problemas alimentares da criança. Instrumentos desse tipo apresentam grande relevância, uma vez que os pais/cuidadores, ao observar inúmeras refeições, podem ter uma perspectiva mais ampla do comportamento alimentar típico da criança. Sendo assim, o relato destes pode ser uma forma de avaliação aplicável e confiável, adicionando informações importantes sobre o comportamento e habilidades típicas na hora da refeição, incluindo tempo de refeição^(12)^.

O presente estudo apresenta como limitações o seu caráter observacional com uso de um instrumento de rastreio, ao invés de avaliações completas dos casos. Cabe salientar que não existe na literatura brasileira, instrumento avaliativo validado para as DAs atualmente. Outra limitação foi ser desenvolvido através de questionário de preenchimento online, o que pode gerar viés de seleção e acabar por selecionar indivíduos de renda e escolaridade maiores. Todavia, os achados do presente estudo contribuem para maior apresentação e entendimento da temática das DAs, que mostraram-se pouco encontradas na literatura, especialmente na nacional. Também, trata-se de um dos poucos estudos de ocorrência na literatura nacional, mostrando-se inovador por utilizar instrumento validado para direcionar a presença ou não, e a severidade das DAs. Desta forma, mais estudos abordando ocorrência, incidência, diagnóstico e direcionando tratamento das DAs fazem-se necessários.

## CONCLUSÃO

Portanto, foi possível estimar a ocorrência de dificuldades alimentares nas crianças brasileiras desse estudo, a partir do preenchimento do instrumento EBAI pelos pais ou responsáveis em 26%. A maioria das crianças com presença de DAs as apresentou com gravidade severa, associada a comorbidades pré-existentes. Houve associação positiva entre a escolaridade e renda familiar e a presença e gravidade da DA.
